# An exploration of the factors affecting the likelihood of young people in England progressing into higher education

**DOI:** 10.23889/ijpds.v8i2.2311

**Published:** 2023-09-14

**Authors:** Paul Martin

**Affiliations:** 1 University of Warwick, Coventry, United Kingdom

## Objectives

This research sought to identify the extent to which the likelihood of young people in England progressing into Higher Education (HE) is affected by their gender, ethnicity, socioeconomic background, area of residence, school attainment and the intersection of these characteristics.

## Methods

Data from the Department for Education’s National Pupil Database concerning the entire cohort of young people in English state schools who turned 16 between September 2014 and August 2015 was accessed. Data concerned pupils’ attainment, personal characteristics and postcode of residence. This data was matched with records from the Higher Education Statistics Agency to identify whether pupils had progressed to HE by the age of 19. Raw differences in progression rates by pupil characteristic were established and binary logistic regression was used to explore how individual factors influenced the likelihood of progression once attainment and other characteristics were controlled for.

## Results

Females were more likely than males to progress to HE, however this could be explained predominantly by their higher average attainment in school examinations. Pupils in receipt of free school meals were substantially less likely to progress to HE, though this trend reversed once attainment was controlled for.

Pupils residing in London were considerably more likely to progress to HE than pupils in all other regions of England, though this can be explained almost entirely by the higher average attainment of pupils in London as well as average demographic differences between London and other English regions.

There are large disparities in progression to HE by pupil ethnicity and these disparities persist even once prior attainment and other characteristics are controlled for.

## Conclusion

The results overall suggest that some disparities in access to HE (such as by gender and FSM status) might be eliminated if corresponding disparities in school attainment were eliminated. The relationship between attainment disparities and HE progression disparities is less straightforward with respect to other characteristics such as pupil ethnicity.

